# Effect of repeated intraperitoneal injections of different concentrations of oxycodone on immune function in mice

**DOI:** 10.3389/fphar.2024.1370663

**Published:** 2024-06-17

**Authors:** Sumeng Chen, Jingjing Liu, Shaoqiang Huang

**Affiliations:** Department of Anesthesia, Obstetrics and Gynecology Hospital of Fudan University, Shanghai, China

**Keywords:** oxycodone, immune function, morphine, opioid receptor, immune cells

## Abstract

**Background:**

The effect of oxycodone as an opioid receptor agonist on immune function is still controversial. In this study, we investigated the possible effects of oxycodone on immune function in mice and its possible mechanisms of action.

**Methods:**

By repeated intraperitoneal injections of 25 mg/kg morphine and 5 mg/kg, 20 mg/kg, and 60 mg/kg oxycodone, we assessed possible changes in the number of splenic lymphocytes and inflammatory cytokines in the serum of mice. CD4^+^ T cells and CD8^+^ T cells were sorted from the spleen to observe whether the expression levels of opioid receptors and downstream signals were altered.

**Results:**

Repeated administration of oxycodone at a dose above 20 mg/kg resulted in significant weight loss. Repeated administration of oxycodone exhibits significant dose-dependent reduction in CD4^+^ T cells, with little effect on CD8^+^ T cells and little effect on inflammatory cytokine levels. Low- and intermediate-dose oxycodone increased the mRNA expression level of MOR, KOR, and DOR to varying degrees. Moreover, oxycodone increases the mRNA expression levels of the TLR4 signaling pathway to varying degrees.

**Conclusion:**

Repeated intraperitoneal injection of oxycodone induces immunosuppression in mice.

## Introduction

Opioids are the foremost and widely employed medications for managing acute intraoperative and postoperative pain, offering notable advantages such as absence of gastrointestinal hemorrhage risk and absence of the analgesic “ceiling effect” observed with non-steroidal anti-inflammatory drugs (NSAIDs) (Plein and Rittner, 2018). Many studies have explored the effects of opioids on immune function, both *in vivo* and *in vitro*. The extent of their influence varies significantly based on factors such as opioid class, dosage, administration method, and treatment regimen (Boland et al., 2014). Among the myriad investigations of opioid effects on immunity, morphine emerges as the most thoroughly examined drug, consistently revealing inhibitory effects on immune function, with a relatively clear mechanism of action (Ninković and Roy, 2013; Feehan and Zadina, 2019; Maher et al., 2020; Jiang et al., 2022).

As one of the most commonly used opioids for treating acute and chronic pain, oxycodone is a relatively selective MOR agonist; it can also bind to KOR and DOR with very low affinity, in the μM range (Peckham and Traynor, 2006; Marie and Noble, 2023). Zhang et al. (2017) observed significant changes in the expression of genes associated with inflammation and immune function upon oxycodone administration, supporting its link to inflammation and immune response. However, controversy persists regarding the precise impact of oxycodone on immune function. While limited studies suggest that oxycodone exerts minimal effects on immunity, notably less than that exerted by morphine or fentanyl (Franchi et al., 2019), an *in vitro* study using mouse splenocytes reported an inhibitory effect of oxycodone on lymphoproliferation, characterized by an inverted bell-shaped curve with only select intermediate concentrations exhibiting activity (Hutchinson and Somogyi, 2004).

The activation of the MAPK and NF-κB signaling pathways by morphine has been confirmed by numerous studies (Ji et al., 2020; Ren et al., 2022; Ahmadi et al., 2023; Yang et al., 2023). However, the role of Toll-like receptor 4 (TLR4) in this process remains highly controversial (Eisenstein, 2019; Zhang et al., 2020). Currently, there is no research investigating the effects of oxycodone on opioid receptors and the aforementioned key inflammatory signaling molecules (including NF-κB, P38, and TLR4) in peripheral immune cells.

This study aimed to investigate whether oxycodone exhibits an immunosuppressive effect similar to that of morphine and whether there are changes in opioid receptors and inflammatory signaling molecules on peripheral T cells.

## Materials and methods

### Animals

Only male C57BL/J6 mice, aged 6–8 weeks and weighing 19–25 g, obtained from Shanghai Jiesijie Laboratory Animal Co., Ltd. (Shanghai, China), were used in all experiments. The mice were housed in a controlled environment with a 12/12-h light/dark cycle (lights on at 8:00 a.m. and off at 8:00 p.m.) and maintained at a temperature of 25°C, with free access to food and water. Animal care and experimental procedures were conducted in compliance with the Guide for the Care and Use of Laboratory Animals (Institute of Laboratory Animal Resources Commission on Life Sciences, 2016). Following 1 week of acclimatization, the animals were randomly assigned into five groups. All experimental protocols involving animals were approved by the Ethics Committee of Animal Experimentation Project, Department of Laboratory Animal Science, Fudan University (Permission number: 202306003Z). Every effort was undertaken to minimize the number of animals used and alleviate any potential suffering throughout the experiments.

### Drug administration

The primary drugs used in this study were morphine hydrochloride (Shenyang First Pharmaceutical Company, Shenyang, China) and oxycodone hydrochloride (Mundipharma Pharmaceutical Co., Ltd., Beijing, China). Both drugs were dissolved in a saline solution. The control group received an equivalent volume of saline as a negative control, the morphine group served as the positive control, and the low-, medium-, and high-dose oxycodone groups were administered oxycodone hydrochloride at doses of 5 mg/kg, 20 mg/kg, and 60 mg/kg body weight, respectively.

**FIGURE 1:**
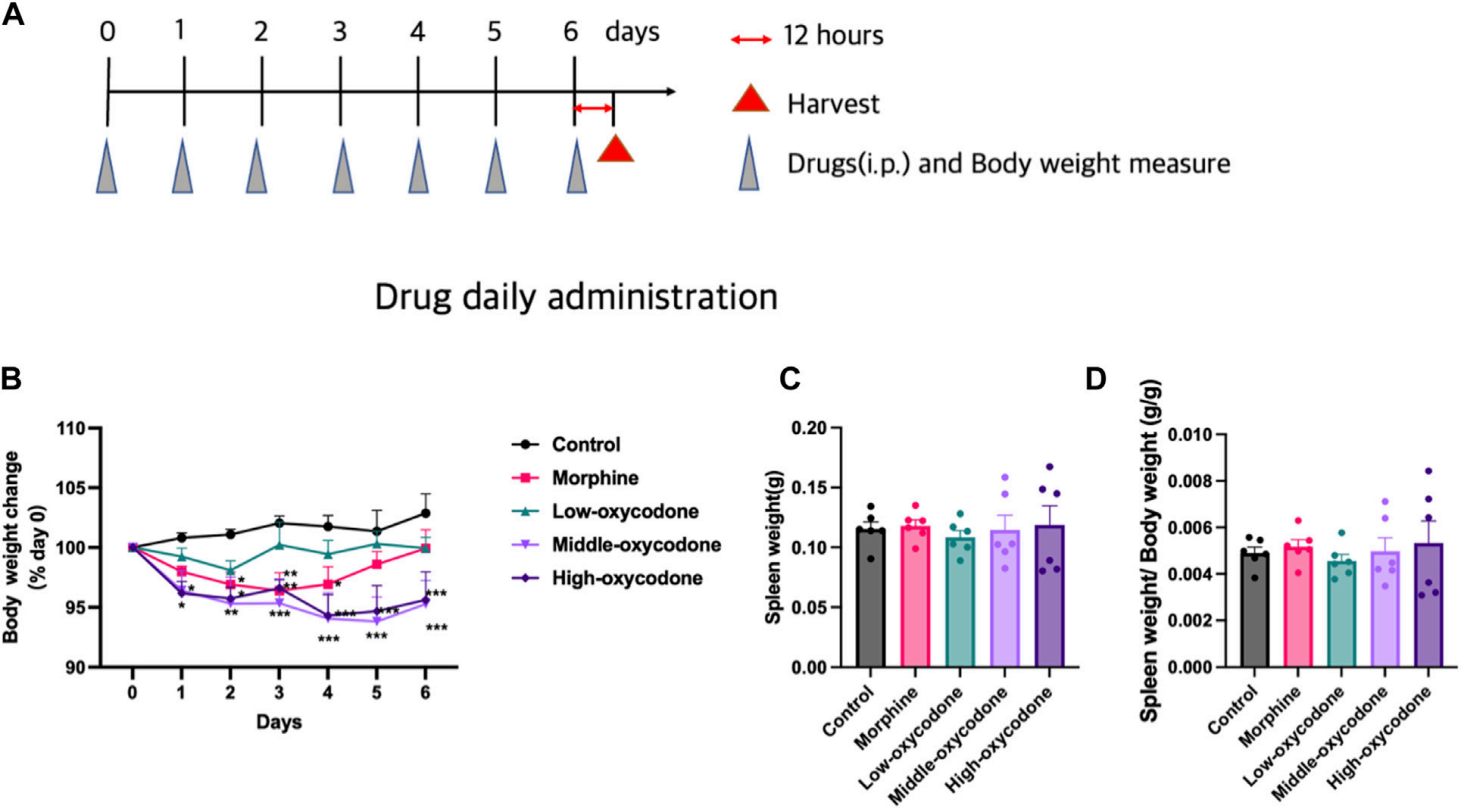
Effects of repeated intraperitoneal injection of drugs on the spleen and body weight. **(A)** Experimental timeline. **(B)** Body weight change in each group (two-way ANOVA test: *p < 0.05, **p < 0.01, and ***p < 0.001 vs. control group). **(C)** Spleen weight in each group. **(D)** Spleen/body weight ratios in each group (one-way ANOVA test: *p < 0.05 and ***p < 0.001 vs. the control group; #*p* < 0.05 and ##*p* < 0.01 vs. the morphine group). The data represent the mean ± SEM of animals (*n* = 6 mice per group).

Different groups of mice were weighed and injected intraperitoneally once daily at a consistent time point over the course of 1 week (Figure 1A).

### Flow cytometry

After 1 week of drug administration, spleens were harvested 12 h after the last injection (Figure 1A), and the body weight at the time of the final injection was utilized to calculate the spleen/body weight ratio (Figures 1C, D). The mice were anesthetized via intraperitoneal injection of sodium pentobarbital for spleen isolation. The spleens were then homogenized with PBS and filtered through a 70-μm cell strainer to obtain a single-cell suspension. Subsequently, red blood cell lysis buffer (Beijing Solarbio Science and Technology Co., Ltd., Beijing, China) was applied to the single-cell suspension of the spleen for hemolysis. For blocking, the cells were treated with an anti-CD16/32 antibody (from BD Biosciences, Inc., Franklin Lakes, NJ, United States). Immune cells were then labeled with specific antibodies (Table 1). The proportions of different immune cell types were analyzed using a Beckman CytoFLEX Flow Cytometry system. CD4^+^ T cells and CD8^+^ T cells were sorted using a Beckman MoFlo Astrios Cell Sorter.

**TABLE 1:** Antibodies used in flow cytometry.

Target cell	Antibody
CD8^+^/CD4^+^ T cell	FITC anti-mouse CD4 antibody (#100405; BioLegend Inc., SD, United States)
	PE anti-mouse CD8a antibody (#100707; BioLegend Inc.)
	APC/cyanine7 anti-mouse CD45.2 antibody (#109824; BioLegend Inc.)
NK cell	APC anti-mouse CD49b (pan-NK cells) antibody (#108909; BioLegend Inc.)
	PE anti-mouse CD3ε antibody (#100307; BioLegend Inc.)
	APC/cyanine7 anti-mouse CD45.2 antibody (#109823; BioLegend Inc.)

### Real-time quantitative reverse-transcription polymerase chain reaction

Total RNAs were extracted using TRIzol (Beyotime Biotech, Co., Ltd., Shanghai, China) from T cells sorted by fluorescence-activated cell sorting. Subsequently, cDNAs were synthesized utilizing the PrimeScript™ RT reagent Kit (Takara Biotechnology, Co., Ltd., Beijing, China). Quantitative measurements were carried out on an Applied Biosystems™ QuantStudio™ 5 Real-Time PCR System (Applied Biosystems, Waltham, MA, United States) employing the Hieff^®^ qPCR SYBR Green Master Mix (Yisheng Biotechnology, Co., Ltd., Shanghai, China), with relative gene mRNA levels normalized to glyceraldehyde 3-phosphate dehydrogenase (GAPDH). The sequences of the primers used are given in Table 2. The mRNA expressions level was calculated using the 2^−ΔΔCt^ method. Each experiment was conducted three times.

**TABLE 2:** Primer sequences used for real-time quantitative reverse-transcription polymerase chain reaction (RT-qPCR).

Symbol	Sequences (5′→3′)
*Gapdh*	Forward: CAT​GGC​CTT​CCG​TGT​TCC​TA
	Reverse: GAT​GCC​TGC​TTC​ACC​ACC​TT
*Oprd1*	Forward: AGC​GTG​GAC​CGC​TAC​ATT​G
	Reverse: CCA​AGA​CCC​AGA​TGC​ATA​TAT​TGA
*Oprk1*	Forward: CCT​TTT​GGA​GAT​GTG​CTA​TGC​A
	Reverse: TGT​AGC​GGT​CCA​CAC​TCA​TCA
*Oprm1*	Forward: GTG​TGT​AGT​GGG​CCT​CTT​TGG
	Reverse: TGC​CAG​AGC​AAG​GTT​GAA​AA
*Tlr4*	Forward: AGG​CAT​GGC​ATG​GCT​TAC​AC
	Reverse: TCT​CCA​CAG​CCA​CCA​GAT​TCT
*P38*	Forward: CACCTGCAAGGTCCCTGG
	Reverse: CAGGTCTGCCCCCATGAG
*NF-κB65*	Forward: CAT​GTC​TCA​CTC​CAC​AGC​T
	Reverse: CCGGAGAGACCATTGGGA

### Enzyme-linked immunosorbent assay

Whole blood was collected, and serum was separated by centrifugation at 3,000 rpm for 15 min at 4°C. The separated serum was then collected and stored at −20°C. Serum levels of interleukin (IL)-2, IL-6, and IL-10 and tumor necrosis factor (TNF)-α were determined using enzyme-linked immunosorbent assay (ELISA) kits (Enzyme-linked Biotechnology Co., Ltd., Shanghai, China) following the manufacturer’s protocol.

### Statistical analysis

The data are presented as the mean ± standard error of the mean (SEM). Flow cytometry and ELISA experiments were conducted at least twice. Statistical analysis was performed using one-way ANOVA followed by Tukey’s *post hoc* test or two-way repeated measure ANOVA followed by Bonferroni’s *post hoc* test, as appropriate. Statistical analysis was carried out using GraphPad Prism (ver9.0; GraphPad Software Inc., San Diego, CA, United States), with differences considered significant at *p* < 0.05.

## Results

### Decreased body weight in mice with oxycodone injection

To assess the impact of oxycodone on mouse body weight and determine any dose-dependent effects, the post-dose body weight was compared to the pre-dose body weight, resulting in a line chart illustrating body weight changes over time. Administration of low-dose oxycodone at 5 mg/kg did not produce any noticeable effect on mouse body weight. However, mice in the morphine group exhibited weight loss on days 1, 2, 3, and 4, followed by a gradual return to nearly pre-dose levels after day 5. In contrast, mice in the medium- and high-dose oxycodone groups (20 mg/kg and 60 mg/kg, respectively) experienced significant weight loss, starting from day 2, and did not recover (Figure 1B). Notably, different drug injections did not exert a significant effect on spleen weight or the spleen/body weight ratio (Figures 1C, D).

### Effect of oxycodone administration on immune cells of the spleen

**FIGURE 2:**
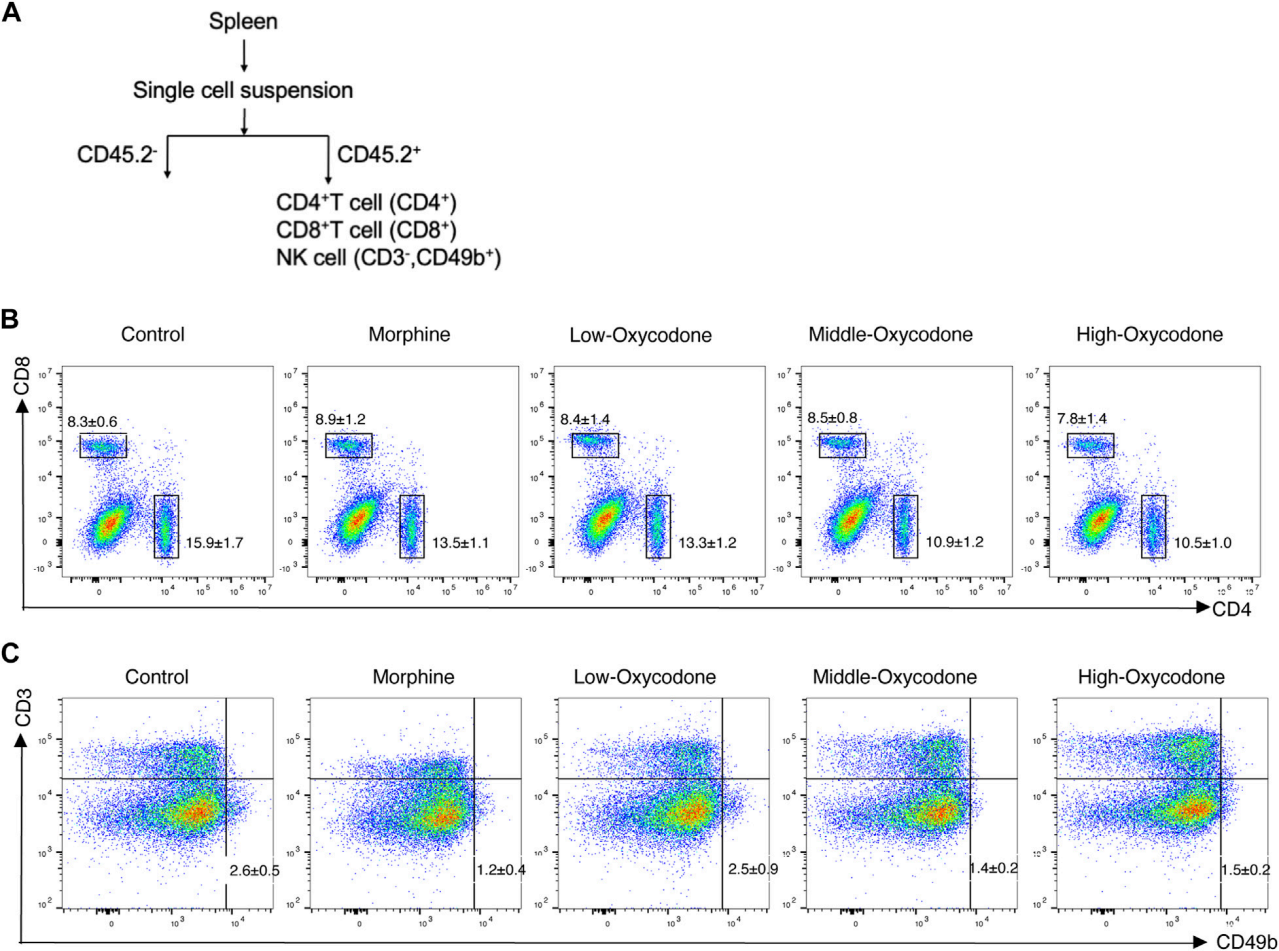
Flow cytometry workflow and representative plots of each group. Schematic workflow of the isolation of splenic leucocytes using flow cytometry **(A)**. Representative flow cytometric plots of CD4^+^ T and CD8^+^ T cells **(B)** and NK cells **(C)** derived from the spleen of mice administered repeatedly with the drug.

To determine whether oxycodone exhibits immunosuppressive effects similar to those of morphine, our investigation centered on its impact on the immune system. Initially, we evaluated alterations in the number of splenic lymphocytes in mice subjected to repeated intraperitoneal injections of oxycodone utilizing flow cytometry (Figure 2).

**FIGURE 3:**
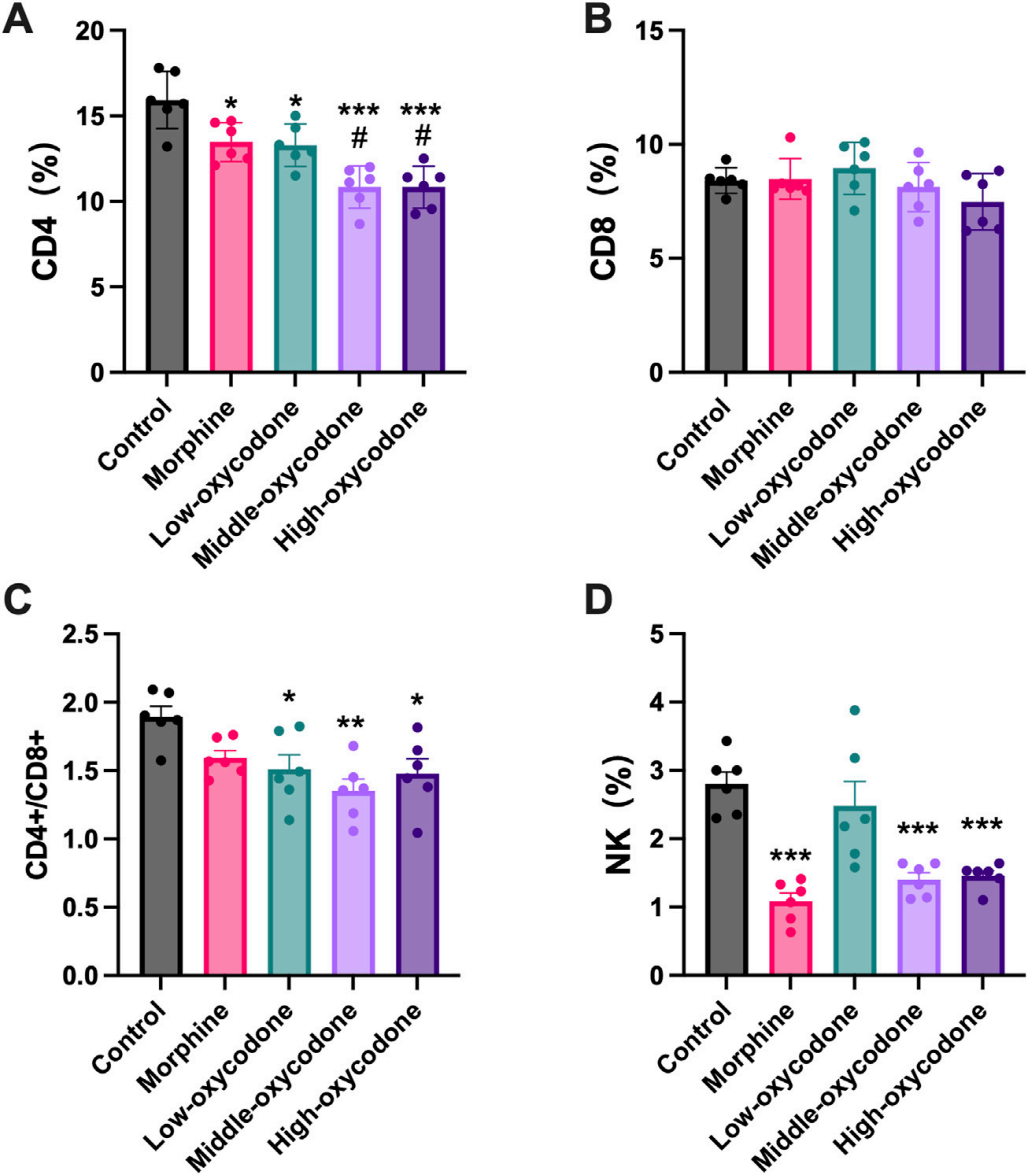
Changes in the number of spleen-derived immune cells by the repeated administration of the drug. Quantitative evaluation of the number of CD4^+^ T cells **(A)**, CD8^+^ T cells **(B)**, CD4^+^ T cells/CD8^+^ T cell ratio **(C)**, and NK cells **(D)** derived from the spleen of mice treated repeatedly with different drugs. Each value represents the mean ± SEM (*n* = 6; one-way ANOVA test: *p < 0.05 and ***p < 0.001 vs. the control group; #*p* < 0.05 and ##*p* < 0.01 vs. the morphine group).

Our findings revealed a decrease in the percentage of splenic CD4^+^ T-cell subsets in the oxycodone groups compared to the control group, with more pronounced reductions observed in the medium- and high-dose oxycodone groups than in the morphine group (Figure 3A). However, repeated intraperitoneal injections of opioids did not exert a significant impact on CD8^+^ T cells (Figure 3B). Furthermore, the CD4^+^ T cell/CD8^+^ T cell ratio was statistically decreased in the oxycodone-administered group (Figure 3C). Morphine, as well as medium- and high-dose oxycodone, significantly inhibited NK cell numbers (Figure 3D). Hence, it can be inferred that repeated intraperitoneal oxycodone injections have an inhibitory effect on immune function. Oxycodone at 20 mg/kg demonstrates suppressive effects, with the drug’s efficacy plateauing, as higher doses do not confer additional immunosuppressive effects.

### Effect of oxycodone administration on mRNA expression in immunological cells of the spleen

**FIGURE 4:**
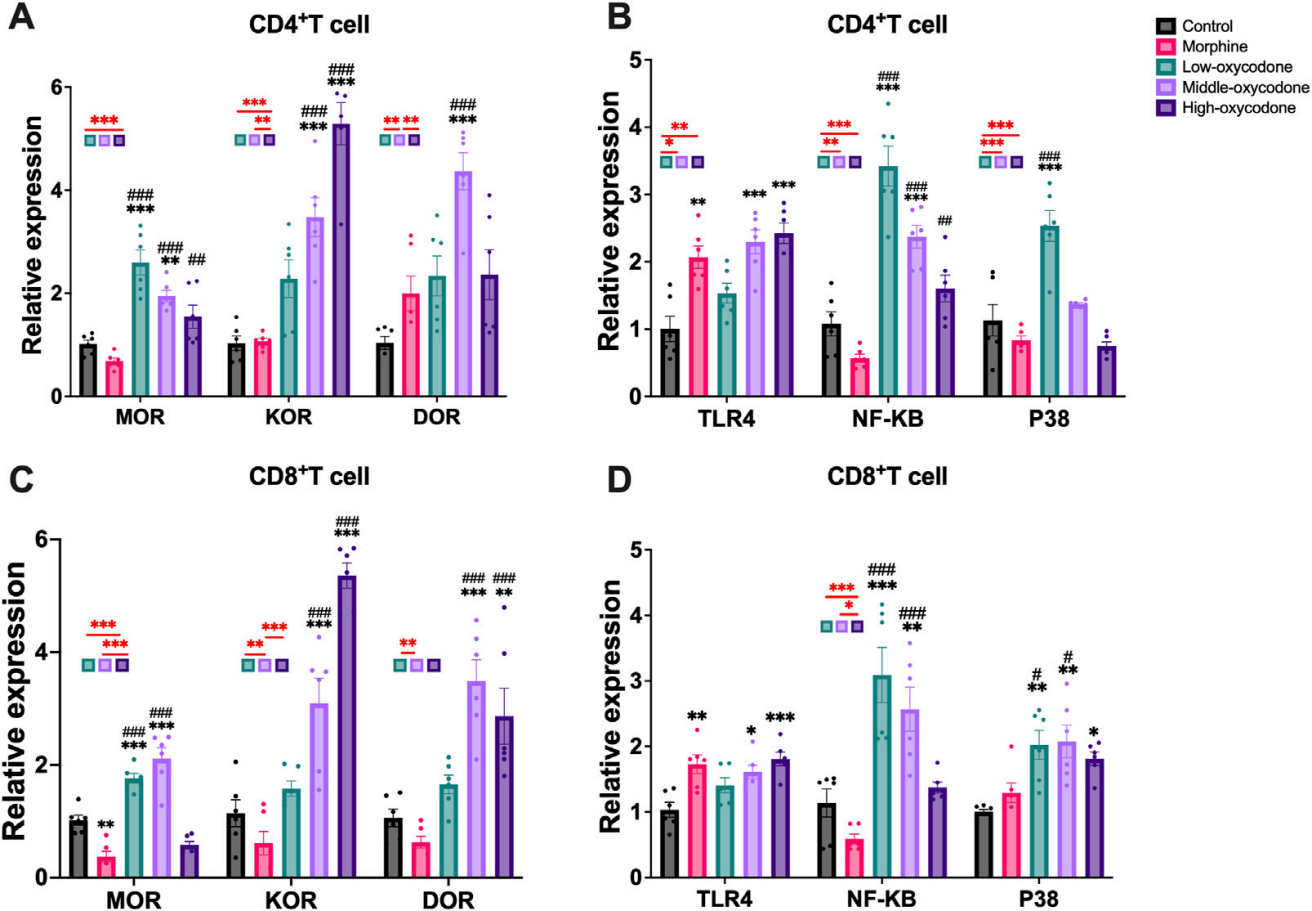
Changes in the mRNA expression of opioid receptors and other related receptors in the CD4^+^ T cells and CD8^+^ T cells sorted from the spleen of mice by repeated intraperitoneal injection of drugs. Changes in the mRNA expression of MOR, KOR, and DOR **(A)** and TLR4, NF-κB, and P38 **(B)** in CD4^+^ T cells. Changes in the mRNA expression of MOR, KOR, and DOR **(C)** and TLR4, NF-κB, and P38 **(D)** in CD8^+^ T cells. Each bar represents the mean ± SEM (*n* = 6; one-way ANOVA test: *p < 0.05, **p < 0.01, and ***p < 0.001 vs. the control group; #*p* < 0.05 and ###*p* < 0.001 vs. the morphine group).

To investigate alterations in the mRNA levels of opioid receptor subtypes (MOR, DOR, and KOR) in different immune cells, we used FACS to sort out CD4^+^ T cells (Figures 4A, B) and CD8^+^ T cells (Figures 4C, D) from the mouse spleen following repeated intraperitoneal opioid injections.

In CD4^+^ T cells, the mRNA level of MOR was significantly increased in the low- and medium-dose oxycodone groups compared to the control and morphine groups. The mRNA level of KOR was similar in the low-dose oxycodone group and the control and morphine groups, but it was significantly and dose-dependently elevated in the medium- and high-dose oxycodone groups. Additionally, the medium- and high-dose oxycodone groups exhibited elevated mRNA levels of DOR. Morphine, as well as the medium- and high-dose oxycodone groups, showed significantly elevated TLR4 mRNA levels. Furthermore, as the concentration of oxycodone increased, NF-κB and P38 mRNA levels gradually decreased. Repeated morphine injection had no significant effect on NF-κB and P38.

In CD8^+^ T cells, repeated administration of morphine did not affect opioid receptor expression. However, KOR mRNA levels gradually increased with increasing concentrations of oxycodone, while the increase in DOR mRNA levels was only observed in the medium-dose oxycodone group. Similar to CD4^+^ T cells, morphine, as well as the medium- and high-dose oxycodone, caused a significant increase in TLR4 levels, and oxycodone injection also elevated the P38 level. Furthermore, oxycodone dose-dependently led to a gradual decrease in NF-κB levels, but this was not correlated with P38 levels.

In summary, our findings indicate that oxycodone can enhance the level of MOR and KOR on the surface of immune cells. The expression of KOR on immune cell surfaces increases with higher concentrations of repeated oxycodone injections, whereas the expression of MOR appears to be independent of the concentration. Oxycodone inhibits immune function and alters the TLR4 and NF-κB mRNA levels on the surface of T cells.

### Effect of oxycodone administration on immune cytokines

**FIGURE 5:**
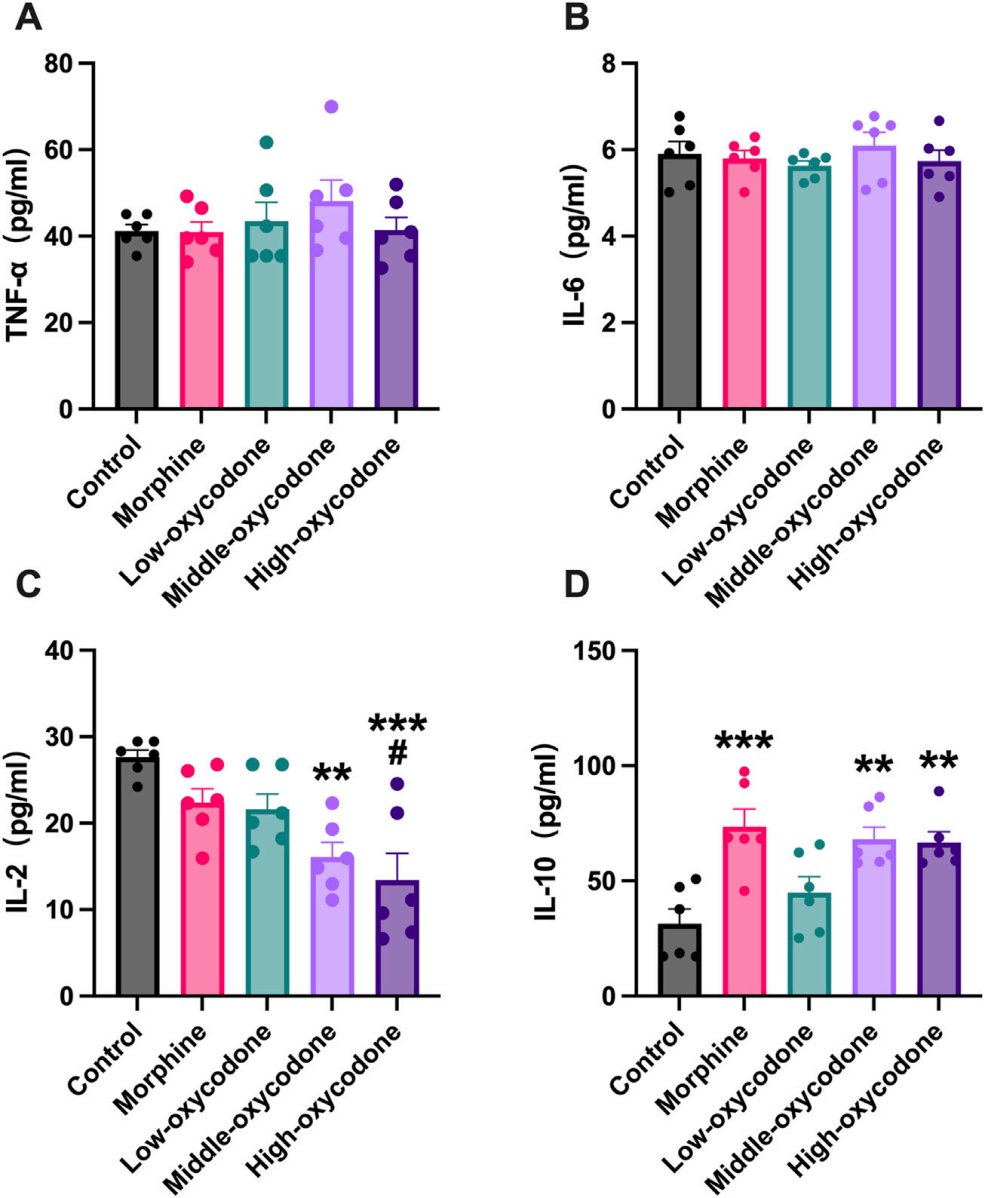
Effect of repeated intraperitoneal injection of drugs on inflammatory cytokine expression in the serum. **(A)** TNF-α, **(B)** IL-6, **(C)** IL-2, and **(D)** IL-10 concentrations in the serum were detected by ELISA. Data are shown as mean ± SEM (*n* = 6; one-way ANOVA test: *p < 0.05, **p < 0.01, and ***p < 0.001 vs. the control group; #*p* < 0.05 vs. the morphine group).

To investigate the impact of oxycodone on the release of inflammatory cytokines, including both pro-inflammatory and anti-inflammatory cytokines, we assessed several typical inflammatory cytokines, including TNF-α (Figure 5A), IL-6 (Figure 5B), IL-2 (Figure 5C), and IL-10 (Figure 5D), using ELISA kits. Our findings suggest that oxycodone has a minimal effect on the release of TNF-α and IL-6 overall. However, the medium- and high-dose oxycodone groups exhibited significantly decreased levels of IL-2. The morphine and medium- and high-dose oxycodone groups demonstrated increased levels of the anti-inflammatory cytokine IL-10.

## Discussion

Oxycodone has found extensive application in clinical practice, particularly in managing cancer pain and postoperative pain. Nonetheless, its impact on immune function and the underlying mechanism remain contentious. Hence, we formulated an experimental protocol to examine the effects of varied oxycodone dosages on immune function and elucidate potential mechanisms. Our findings revealed that oxycodone has the capacity to suppress the percentages of CD4^+^ T cells and NK cells. Additionally, oxycodone demonstrated an ability to enhance the mRNA levels of MOR and KOR, with a notable increase observed in KOR levels, correlating with escalating oxycodone doses. Moreover, oxycodone elevated the mRNA levels of TLR4 and NF-κB in T cells. Notably, medium- and high-dose oxycodone regimens were found to reduce IL-2 levels while increasing IL-10 levels in serum, further highlighting its immunomodulatory effects.

The occurrence of weight loss may be attributed to restlessness and increased movement observed in mice following repeated opioid injections. Prior to drug administration, there was minimal disparity in the motor status of mice in the control and low-dose oxycodone groups, with all exhibiting relative calmness. However, following the administration of morphine and medium- and high-dose oxycodone, mice displayed heightened circling behavior in their cages, a noticeable inclination toward agitation, and occasional instances of fighting behavior. Nevertheless, these behavioral abnormalities were generally normalized before the subsequent day’s injection. This finding aligns with the study findings obtained by Koek (2014), who noted a pattern of initial weight loss, followed by recovery in adult mice after morphine administration. Additionally, previous research has reported that repeated injections of oxycodone lead to increased jumping behavior and decreased body weight (Scicluna et al., 2022).

In numerous experiments, doses equal to or greater than 15 mg/kg of morphine have been shown to induce immunosuppression (Nelson et al., 1997; Limiroli et al., 2002; Massi et al., 2003). Lysle et al. (1993) reported significant immune function suppression in rats administered with morphine at doses of 15 mg/kg and 25 mg/kg, with no notable difference between the two doses. Therefore, to ensure a more pronounced immunosuppressive effect as a positive control, we selected the dose of 25 mg/kg. In rats, oxycodone exhibits an analgesic potency 2–4 times that of morphine after systemic administration (subcutaneous or intraperitoneal) (Pöyhiä et al., 1992; Pöyhiä and Kalso, 1992; Lemberg et al., 2006; Yang et al., 2016). Additionally, one human study found that the equivalent analgesic dose of oxycodone was approximately two-thirds of morphine (Pöyhiä et al., 1992). Thus, we estimated the equivalent analgesic dose of oxycodone corresponding to 25 mg/kg morphine to be 6.25–16.5 mg/kg, with a significant difference between the lower and upper bounds. Consequently, we chose 5 mg/kg and 20 mg/kg as the low- and medium-dose oxycodone groups, respectively. Previous mouse experiments have utilized oxycodone doses ranging from 0.5 mg/kg to 100 mg/kg, depending on experimental objectives (Madia et al., 2009; Carper et al., 2021). However, doses exceeding 60 mg/kg in mice may result in animal death (Henningfield et al., 2022). To minimize unnecessary animal death and tolerance, we ultimately set the high oxycodone dose to 60 mg/kg.

Our study revealed that the impacts of repeated opioid injections on T cells were primarily focused on CD4^+^ T cells, with low doses of oxycodone (5 mg/kg) eliciting similar inhibitory effects as morphine (25 mg/kg). Repeated intraperitoneal opioid injections did not influence CD8^+^ T cells. Furthermore, NK cells were reduced in all groups, except the low-dose oxycodone group. It can be concluded that oxycodone has an immunosuppressive effect in both innate and adaptive immunity. In previous clinical trials, oxycodone was often regarded as either not affecting immune function or mildly suppressing it. In one human study (Cui et al., 2017), the postoperative intravenous injection of 5 mg oxycodone resulted in a decrease in T cells and NK cells, but the decrease in CD8^+^ T cells compared to that of CD4^+^ T cells was not as pronounced, which is similar to the results of this study. Although this study involved a single dose and only observed changes within 24 h post-administration, it still provides supporting evidence for our conclusion to a certain extent. The morphine dose we used is roughly equivalent to 4 mg/kg in humans (adjusted by dividing by 6.2 for allometric scaling). Calculated for an average adult weight of 60 kg, this translates to approximately 240 mg/day. Wiffen et al. (2013) stated that daily morphine doses for cancer pain management ranged from 25 mg to 2,000 mg with an average of between 100 mg and 250 mg. The oxycodone dose was determined based on equianalgesic doses of morphine. Therefore, the dose of the drug is clinically relevant. Based on our experimental design, the results of this study are more likely to be applicable to patients who use opioid medications in the long term, such as patients with advanced cancer pain or individuals with drug use disorders. Both of these patient populations already experience immune suppression issues. Therefore, even a 3%–5% reduction in CD4^+^ T cells, as observed in our study, holds clinical significance for these patients.

To investigate whether oxycodone affects the release of inflammatory cytokines, we assessed several typical cytokines, including pro-inflammatory cytokines like TNF-α, IL-2, and IL-6, and the anti-inflammatory cytokine IL-10. Our findings indicated that neither morphine nor oxycodone influenced the expression of TNF-α and IL-6. This outcome could be attributed, in part, to the short duration of drug administration as the 1-week time frame may not have been sufficient to significantly impact inflammatory cytokine levels. Additionally, it could also be related to the fact that we collected blood samples 12 h after the last injection. The observed elevation in IL-10 levels with morphine administration alone could be linked to the duration of drug exposure in our study. A prior investigation (Sacerdote, 2003) found that the impact on IL-12 disappeared by day 3 in mice subjected to prolonged morphine administration, while the influence on IL-10 persisted until day 12.

The medium- and high-dose oxycodone groups exhibited a significant decrease in IL-2 levels, whereas the morphine and medium- and high-dose oxycodone groups showed an increase in the anti-inflammatory cytokine IL-10 levels. Weber and Pert (1989) discovered that morphine injected into the periaqueductal gray (PAG) significantly suppressed splenic NK cell and T-cell functions by reducing the ability of T cells to produce IL-2. At steady state, IL-2 is produced mainly by CD4^+^ T cells and, to a lesser extent, by CD8^+^ T, NK, and dendritic cells (DCs). Some studies (Amado et al., 2013; Coppola et al., 2020) stipulated that there was a positive correlation between the growth rate of CD4^+^ T cells and the presence of IL-2 and IL-7. Therefore, the decrease in IL-2 levels may be related to the decrease in CD4^+^ T cells. Although there was no statistically significant difference in IL-2 levels between the morphine group and the low-dose hydrocodone group, the IL-2 levels in these two groups were still slightly lower than those in the control group. IL-10 is produced by macrophages, DCs, B cells, various subsets of T cells, and NK cells themselves. Several diverging effects of IL-10 on NK cells have been described. Most of these effects seem to be indirect; indeed, IL-10 *in vitro* treatment of purified NK cells does not have noticeable effects (Marçais et al., 2013).

Several studies (Brejchova et al., 2020; Machelska and Celik, 2020; Maher et al., 2020) have demonstrated that opioid receptors are expressed in various immune cells, including T cells, B cells, and macrophages. Consequently, these cells are susceptible to opioid stimulation, which appears to be responsible for opioid-induced immunomodulatory effects (Filipczak-Bryniarska et al., 2018). Mellon and Bayer (1998) confirmed that the activation of central opioid receptor subtypes by morphine results in changes in peripheral lymphocyte activity that can be mediated through distinct peripheral mechanisms. Pharmacological and pharmacogenomic research has provided evidence that the activation of peripheral MOR mediates immunosuppressive effects in the peripheral immune system (Suzuki et al., 2000; Campana et al., 2010). However, there is still limited research on whether oxycodone alters opioid receptors and inflammation-related mediators in the peripheral immune system.

We observed that the effects of repeated opioid injections on CD4^+^ T cells and CD8^+^ T cells were not entirely consistent, so we investigated whether the expression of different types of opioid receptors on the peripheral T-cell surface will be different. Subsequently, we collected CD4^+^ T cells and CD8^+^ T cells separately to determine any differences in surface opioid receptor expression. Our findings revealed that repeated morphine injections only inhibited the mRNA level of MOR on CD4^+^ T cells, with no effect on CD8^+^ T cells. This indicates that morphine selectively decreased MOR mRNA levels on peripheral CD4^+^ T cells, a result that deviates from those of previous studies (Brejchova et al., 2020). However, one *in vitro* study (Prenus et al., 2012) also confirmed that chronic morphine treatment leads to the downregulation of MOR gene expression, and the regulation of MOR gene expression is cell-type specific. Additionally, morphine did not affect the expression of KOR and DOR on either CD4^+^ T cells or CD8^+^ T cells. On the other hand, oxycodone generally enhances the mRNA levels of MOR and KOR, with KOR expression increasing gradually with escalating oxycodone doses. Notably, only the 20-mg/kg oxycodone dose exhibited a promoting effect on DOR expression in both CD4^+^ T cells and CD8^+^ T cells. Oxycodone and morphine both reduce CD4^+^ T cell expression, but their effects on the surface molecules of CD4^+^ T cells are inconsistent. We did not find any potential mechanisms in previous literature, so the specific reasons await further investigation.

It must be acknowledged that T cells are not major expressers of TLR4. TLR4 is mainly expressed in monocytes and macrophages. In human studies, Liu et al. (2013) showed a markedly higher expression of TLR4 by PBMCs, CD4^+^ T cells, or monocytes obtained from Behcet’s disease patients than that in controls. Another study also showed increased TLR4 expression on B cells, T cells, granulocytes, and classical monocytes (van der Houwen et al., 2020). Across these studies, mononuclear cells and T cells consistently exhibited parallel alterations. Moreover, numerous investigations have explored the impact of TLR4 on the immune function of T cells (Lee et al., 2017; Gondoh et al., 2022). For example, Du et al. (2022) focused on the role of TLR2/TLR4-mediated activation of CD4^+^ T cells. Although our study did not directly investigate changes in the TLR4 mRNA level of macrophages, alterations on T cells remain meaningful.

To determine whether oxycodone affects immune function through mechanisms similar to those of morphine, we also investigated changes in TLR4, NF-κB, and P38 mRNA levels in T cells. Our results revealed that the repeated administration of both morphine and oxycodone increased the mRNA level of TLR4 receptors on the surface of T cells. However, morphine did not affect the level of NF-κB and P38. Interestingly, Roy et al. demonstrated that murine peritoneal macrophages treated with micromolar concentrations of morphine exhibited decreased NF-κB levels, whereas nanomolar concentrations of morphine led to NF-κB activation (Roy et al., 1998). Therefore, the lack of a statistically significant change in NF-κB mRNA level observed in the morphine group may be attributed to the dose of morphine falling within a concentration range that does not affect NF-κB expression levels. Conversely, oxycodone upregulated the expression of both TLR4 and NF-κB. Interestingly, the level of TLR4 appeared to be independent of the drug dosage, whereas the level of NF-κB decreased gradually with increasing oxycodone doses. This variation may suggest a concentration-related effect of oxycodone on targeting NF-κB. We observed a more pronounced enhancing effect of P38 on CD8^+^ T cells. These findings collectively suggest that oxycodone inhibits immune function and increases the mRNA levels of TLR4 and NF-κB on T cells.

This study has some limitations. First, to evaluate changes in immune function in mice following repeated opioid injection, we employed a once-daily injection regimen for 7 consecutive days. However, we overlooked the possibility that mice receiving repeated opioids might develop drug withdrawal syndrome (Rahim et al., 2002; Feng et al., 2006). Moreover, in order to avoid the transient changes in immune function after drug administration, we collected blood samples and spleen tissue 12 h after the final injection. Compared to other experimental designs where sampling occurs 1 h post-injection, the time interval between the last drug administration and sampling was relatively prolonged in our study. Our study design may better reflect the long-term effects of opioids on immune function following repeated administration. Future experiments could consider sampling at shorter intervals after administration. Second, while changes in mRNA levels of opioid receptors on immune cells were detected, protein expression verification was not performed. Therefore, our study can only speculate on potential mechanisms underlying the emergence of immune suppression by oxycodone, warranting further experimental validation. Finally, only male animals were selected for this study, and female animals were not included. Previous research on oxycodone and other opioid drugs has largely focused on the effects of analgesia and drug abuse, with little evidence of the impact of gender differences on immune function. The conclusion of this study only applies to male mice.

In conclusion, this study examined the effects of repeated injections of various doses of oxycodone compared to morphine on the immune system. The findings suggest that oxycodone induces immunosuppression in mice.

## Data Availability

The raw data supporting the conclusion of this article will be made available by the authors, without undue reservation.
